# Regulation of HCN Channels by Protein Interactions

**DOI:** 10.3389/fphys.2022.928507

**Published:** 2022-06-20

**Authors:** Colin H. Peters, Rohit K. Singh, John R. Bankston, Catherine Proenza

**Affiliations:** ^1^ Department of Physiology and Biophysics, University of Colorado Anschutz Medical Campus, Aurora, CO, United States; ^2^ Department of Cardiology, University of Colorado Anschutz Medical Campus, Aurora, CO, United States

**Keywords:** HCN channels, accessory proteins, protein subunits, TRIP8b, IRAG, KCNE

## Abstract

Hyperpolarization-activated, cyclic nucleotide-sensitive (HCN) channels are key regulators of subthreshold membrane potentials in excitable cells. The four mammalian HCN channel isoforms, HCN1-HCN4, are expressed throughout the body, where they contribute to diverse physiological processes including cardiac pacemaking, sleep-wakefulness cycles, memory, and somatic sensation. While all HCN channel isoforms produce currents when expressed by themselves, an emerging list of interacting proteins shape HCN channel excitability to influence the physiologically relevant output. The best studied of these regulatory proteins is the auxiliary subunit, TRIP8b, which binds to multiple sites in the C-terminus of the HCN channels to regulate expression and disrupt cAMP binding to fine-tune neuronal HCN channel excitability. Less is known about the mechanisms of action of other HCN channel interaction partners like filamin A, Src tyrosine kinase, and MinK-related peptides, which have a range of effects on HCN channel gating and expression. More recently, the inositol trisphosphate receptor-associated cGMP-kinase substrates IRAG1 and LRMP (also known as IRAG2), were discovered as specific regulators of the HCN4 isoform. This review summarizes the known protein interaction partners of HCN channels and their mechanisms of action and identifies gaps in our knowledge.

## Introduction

Hyperpolarization-activated cyclic nucleotide-sensitive (HCN1-4) channels are members of the superfamily of voltage-gated ion channels. HCN channels are structurally similar to voltage-gated K^+^ channels: They are tetramers with each subunit having six transmembrane spanning domains including a positively-charged S4 segment that acts as the voltage sensor. However, HCN channels have the unique properties of activation in response to membrane hyperpolarization, mixed Na^+^ and K^+^ permeability, and regulation by cyclic nucleotides, which were first identified as attributes of the funny current (I_f_) that is produced by HCN channels in cardiac pacemaker cells (Brown et al., 1979; DiFrancesco, 1981; DiFrancesco and Tortora, 1991). These unusual properties allow the channels to regulate a diverse set of physiological functions in cells throughout the body. For example, I_f_ in sinoatrial node myocytes is critical for cardiac pacemaking (DiFrancesco, 2010; Peters et al., 2020b). In the brain, HCN channels are the molecular correlate of the hyperpolarization-activated current (I_h_) that regulates input resistance, pre- and post-synaptic properties, and rhythmic oscillatory patterns (Wahl-Schott and Biel, 2008). And, currents mediated by HCN channels in the gastrointestinal tract regulate peristalsis and mobility (Fisher et al., 2018b; Fujii et al., 2020).

**FIGURE 1:**
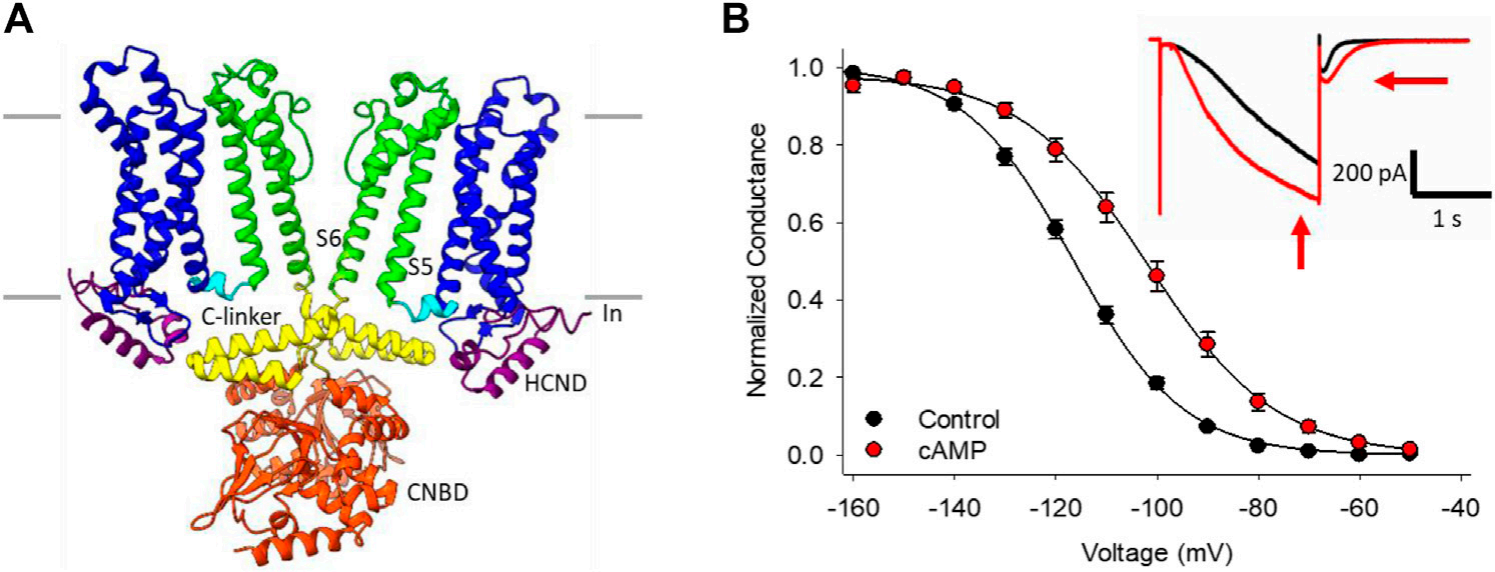
HCN channel structure, currents and regulation by cAMP. **(A)** CryoEM structure of HCN4 showing only two of the four subunits for clarity (PDB ID:6YGO). The six transmembrane-spanning domains in each subunit form the voltage sensing domain (S1-S4, *blue*) and the pore domain (S5-S6, *green*). The C-linker (*yellow*) and cyclic nucleotide binding domain (CNBD, *orange*) in the C-terminus mediate the response to cyclic nucleotides. **(B)** Conductance-voltage relationships for HCN4 channels in the absence (*black*) or presence (*red*) of cAMP. Hyperpolarization-activated currents recorded from a HEK cell expressing HCN4 before (*black*) and after (*red*) perfusion of 100 µM cAMP (*inset*).

The best known HCN channel regulator is cAMP, which binds to a conserved cyclic-nucleotide binding domain (CNBD) in the distal C-terminus of the channel (Figure 1A). In the HCN2 and HCN4 isoforms, cyclic nucleotide binding potentiates the channels by shifting the voltage-dependence of activation by 15–20 mV to more depolarized membrane potentials, speeding the rate of channel activation, and slowing deactivation through distinct mechanisms (Wainger et al., 2001; Wicks et al., 2011; Figure 1B). Unlike HCN2 and HCN4, the HCN1 isoform is minimally sensitive to cyclic nucleotides (∼5 mV shift in voltage dependence), while HCN3 is insensitive (Wainger et al., 2001; Stieber et al., 2005; Xu et al., 2010). cAMP-dependent potentiation of HCN channels is implicated in many physiological contexts from the sympathetic nervous system “fight-or-flight” increase in heart rate (Brown et al., 1979; DiFrancesco, 2010) to the regulation of rhythmic breathing rate (Hawkins et al., 2015; Zhang et al., 2019).

In contrast to the depth of mechanistic knowledge about cAMP regulation (e.g., Porro et al., 2020b; Xia and Accili, 2021), comparatively little is known about how the growing list of protein interaction partners and auxiliary subunits of HCN channels act to alter channel expression, gating, and cAMP-dependent potentiation. This review summarizes the existing information and identifies gaps in knowledge about HCN channel interaction partners.

## TRIP8b

The neuronal tetratricopeptide repeat–containing Rab8b-interacting protein, TRIP8b, is the best-studied of the HCN channel regulatory proteins. Studies have largely defined how TRIP8b interacts with and modulates HCN channels. These data are not only critical for understanding TRIP8b′s role in regulating neuronal excitability, but also as a mechanistic framework for consideration of other HCN interaction partners about which less information is currently available.

**FIGURE 2:**
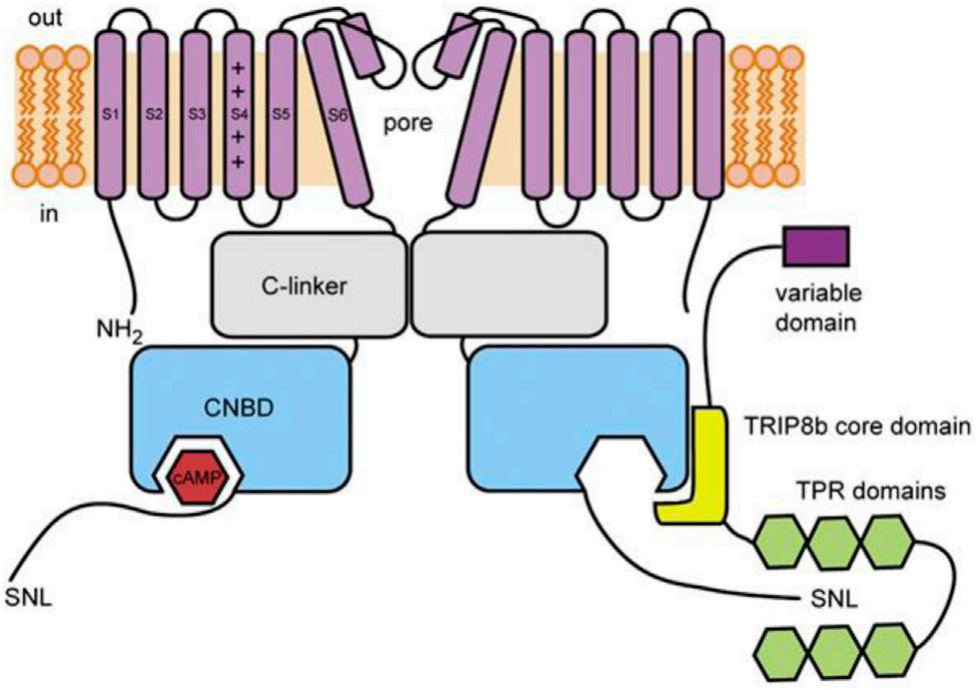
Schematic illustration of TRIP8b and its interaction with HCN channels. The core domain of TRIP8b (*yellow*) antagonizes cAMP binding to the CNBD of HCN channels (*blue*). The TRP domains (*green*) interact with the SNL motif in the distal C-terminus to regulate HCN channel expression. From DeBerg et al., 2015.

TRIP8b (encoded by the peroxisomal biogenesis factor 5 like gene, PEX5L) is a neuron-specific protein (Chen et al., 2001) that was first identified as an HCN channel interaction partner in yeast 2-hybrid screens of a brain library with the C-terminus of HCN channels (Santoro et al., 2004). TRIP8b is a ∼70 kDa protein that contains a series of six tetratricopeptide repeat (TPR) domains, which is a common protein-protein interaction motif. TRIP8b interacts with HCN channels at two distinct sites that have different effects on channel function. As discussed in more detail below, the interaction of TRIP8b with the SNL sequence in the extreme distal C-terminus alters channel trafficking and the interaction with the CNBD inhibits cAMP-dependent potentiation (Lewis et al., 2009; Santoro et al., 2009, 2011; Zolles et al., 2009; Hu et al., 2013; DeBerg et al., 2015; Porro et al., 2020a; Figure 2). Importantly, due to the relative conservation at these sites, TRIP8b interacts with and regulates all four mammalian HCN channel isoforms, albeit with lower affinity in the case of HCN3 (Santoro et al., 2004; Zolles et al., 2009; Han et al., 2011, 2015; Cao-Ehlker et al., 2013; Saponaro et al., 2018).

The interaction of the TPR domains of TRIP8b with the distal C-terminus of HCN channels involves multiple TPR domains within TRIP8b, resulting in a very high affinity interaction (Bankston et al., 2012). These interactions alter channel expression in a complex manner that differs between TRIP8b splice variants. While TRIP8b was originally found to decrease HCN channel trafficking (Santoro et al., 2004), its effects *in vivo* depend on variable alternative splicing of the TRIP8b N-terminus in different regions of the brain (Lewis et al., 2009; Santoro et al., 2009). In the case of HCN1, some TRIP8b variants cause nearly complete internalization while others increase channel expression by 5–10 fold. The interpretation of these experiments is complicated by the lack of a clear relationship between expression of individual exons and HCN1 channel current amplitude. For example, while exons 2 and 4 in the variable N-terminal region contain potential tyrosine and dileucine base trafficking motifs, respectively (Lewis et al., 2009; Santoro et al., 2009), TRIP8b(1b-2) (which contains exon 2 in the exon 1b background) causes a decrease in expression, while TRIP8b(1a-2) does not affect channel expression, and TRIP8b(1a-2–4) increases channel expression. Even more vexing, the effect of different splice variants appears to depend on the expression system. For example, TRIP8b(1b-2–4) increases HCN1 current density in HEK293 cells (Lewis et al., 2009) but decreases current density in *Xenopus laevis* oocytes (Santoro et al., 2009). The picture is likely to be equally complicated in neurons. Knockdown or knockout of all TRIP8b isoforms in the hippocampus results in a loss of proper HCN1 channel localization to the distal dendrites of CA1 neurons with redistribution of HCN1 to axons (Lewis et al., 2011; Piskorowski et al., 2011). A similar pattern was observed when the C-terminal TRIP8b binding site in HCN1 was deleted (HCN1ΔSNL) (Piskorowski et al., 2011). When exons 1b and 2 are knocked out, exons 1a and 1a-4 are sufficient to restore proper channel localization (Piskorowski et al., 2011; Wilkars et al., 2012). In particular, TRIP8b(1a) suppresses HCN1 expression in axons, while TRIP8b(1a-4) enhances HCN1 expression in dendrites (Piskorowski et al., 2011; Wilkars et al., 2012). In contrast, the overexpression of TRIP8b(1b-2) alone acts as a dominant negative disruptor of HCN1 channel expression on the membrane (Fisher et al., 2018a). Unfortunately, less common splice variants of TRIP8b remain understudied and the lack of consistent effects of TRIP8b splice variants on HCN channel expression *in vitro* complicates our understanding of the physiological impacts of TRIP8b on native I_h_ currents. Furthermore, outstanding questions remain about the mechanism of action of the TPR domain interaction with HCN channels.

The interaction of TRIP8b with the CNBD of HCN channels is independent of the variable N-terminus of TRIP8b: all splice variants of TRIP8b similarly antagonize cAMP-dependent-potentiation of HCN channels (Lewis et al., 2009; Santoro et al., 2009). This antagonism is due to the interaction between the 80-amino acid TRIP8b core domain and the conserved HCN channel CNBD (Lewis et al., 2009; Zolles et al., 2009; Santoro et al., 2011; Figure 2). In HCN2 and HCN4, TRIP8b expression reduces the effects of cAMP on the voltage-dependence and rate of activation. In HCN2, TRIP8b also reduces the cAMP-dependent increase in maximal current amplitude (Zolles et al., 2009; Hu et al., 2013; Saponaro et al., 2018). In HCN1, which is thought to be partially activated at basal cAMP concentrations, the effect of TRIP8b is apparent as a hyperpolarizing shift in the basal voltage dependence that is eliminated by mutation of CNBD residues involved in cAMP binding (Santoro et al., 2009; Saponaro et al., 2018). TRIP8b also appears to antagonize the cAMP-dependent slowing of deactivation—it speeds deactivation in HCN1 (Santoro et al., 2009) and appears to prevent cAMP-dependent slowing of deactivation in other HCN isoforms (Hu et al., 2013), although this effect has not yet been quantified.

TRIP8b preferentially binds to the cAMP-free CNBD, and increases in cAMP and TRIP8b have antagonistic effects on binding, however early studies disagreed whether this is due to a direct competition at the binding site or an allosteric mechanism (Han et al., 2011; Hu et al., 2013; Saponaro et al., 2014). Mutation of the cAMP-binding domain arginine (HCN1 R538E) lowers the binding affinity of TRIP8b, suggesting a direct competition (Han et al., 2011). However, initial NMR spectroscopy suggested that TRIP8b primarily interacts with residues in the C-helix and between the E’ helix and A helix, a region termed the N-bundle loop (Saponaro et al., 2014). The authors suggested that interaction with the C-helix stabilizes the cAMP-unbound state without directly competing for the cAMP binding site (Saponaro et al., 2014). Subsequent electron-spin labelling and NMR partially supported the latter conclusion by showing that TRIP8b primarily binds the C-helix and that the TRIP8b-bound CNBD more closely resembles a cAMP-free conformation (DeBerg et al., 2015); however, the data also suggested an interaction of TRIP8b with residues important for cAMP binding and therefore did not rule out a direct competition. Later, a multistate binding model using fluorescence anisotropy data, biolayer interferometry data, and double electron-electron resonance (DEER) supported a mechanism of partial competition for the cAMP binding site (Bankston et al., 2017). This model showed that an 100-fold reduction in the binding affinity for cAMP in the presence of TRIP8b was both necessary and sufficient to explain the reduction in cAMP efficacy on the channel. However, the work by Bankston *et al.* does not rule out a second allosteric mechanism. An NMR structure of a reduced 40 residue TRIP8b core domain (TRIP8b_nano_) coupled with molecular docking and functional analysis of binding site mutants confirmed that TRIP8b directly competes at the cAMP binding pocket and makes additional interactions with the C-helix and N-bundle loop of the CNBD (Saponaro et al., 2018). In addition to stabilizing the binding of TRIP8b to the CNBD (Porro et al., 2020a), the N-bundle loop site could act allosterically to antagonize cAMP (Saponaro et al., 2014; Lyman et al., 2017). These studies all converge on the idea that TRIP8b both shares a number of critical interacting residues with cAMP, but also binds to residues in the N-bundle loop and C-helix that undergo a dramatic rearrangement upon cAMP binding (Saponaro et al., 2014, 2018; DeBerg et al., 2015; Bankston et al., 2017).

While the mechanisms by which TRIP8b reduces the cAMP sensitive of HCN channels are relatively well resolved, the net physiological impacts of TRIP8b on I_h_ in different brain regions remain poorly understood. The role of HCN channels in different neurons, circuits, and behaviours is unclear to begin with and is further complicated by the variable expression and effects of TRIP8b splice variants, which, as described above, can have a net inhibitory or excitatory effect on HCN channels. Thus, the effects of TRIP8b on HCN channels likely differ in different brain regions. Data so far suggest that TRIP8b regulates HCN channels in hippocampal and thalamocortical circuits by controlling maximal expression of HCN channels in CA1 hippocampal neurons and thalamocortical neurons (Santoro et al., 2009; Lewis et al., 2011; Heuermann et al., 2016; Zobeiri et al., 2018). TRIP8b further controls neuronal excitability by regulating trafficking and localization of HCN channels in dendrites (Santoro et al., 2009; Lewis et al., 2011; Piskorowski et al., 2011; Wilkars et al., 2012; Han et al., 2017).

At the behavioural level, TRIP8b expression in CA1 neurons has been linked to major depressive disorder (MDD) and human MDD patients and animal models of chronic stress exhibit higher expression of hippocampal HCN1 (Han et al., 2017; Kim et al., 2018; Lyman et al., 2021). Similarly, while acute cAMP upregulation can reduce spatial memory and motivated behaviours, chronically-elevated cAMP can increase motivated behaviours, possibly through down-regulation of TRIP8b (Lyman et al., 2021). And TRIP8b knockout in mice contributes to increases in motivated behaviours, including more time spent mobile in forced swim and tail suspension tests (Lewis et al., 2011; Han et al., 2017). Thus far, TRIP8b has not been directly implicated in depression in humans, however, as noted in a prior review, studies primarily focus on genes associated with increases in depression so that a lack of direct evidence for anti-depressant activity of TRIP8b is not surprising (Han et al., 2020). In thalamocortical circuits, TRIP8b knockout leads to altered firing patterns associated with transitions between sleep and wakefulness (Zobeiri et al., 2018). And knockout of TRIP8b may predispose mice to absence seizures, while TRIP8b interactions with HCN1 are disrupted by status epilepticus (Shin et al., 2008; Heuermann et al., 2016). Ultimately, the neurological impacts of TRIP8b expression are unlikely to be fully resolved until HCN physiology itself is better understood.

## IRAGs

**FIGURE 3:**
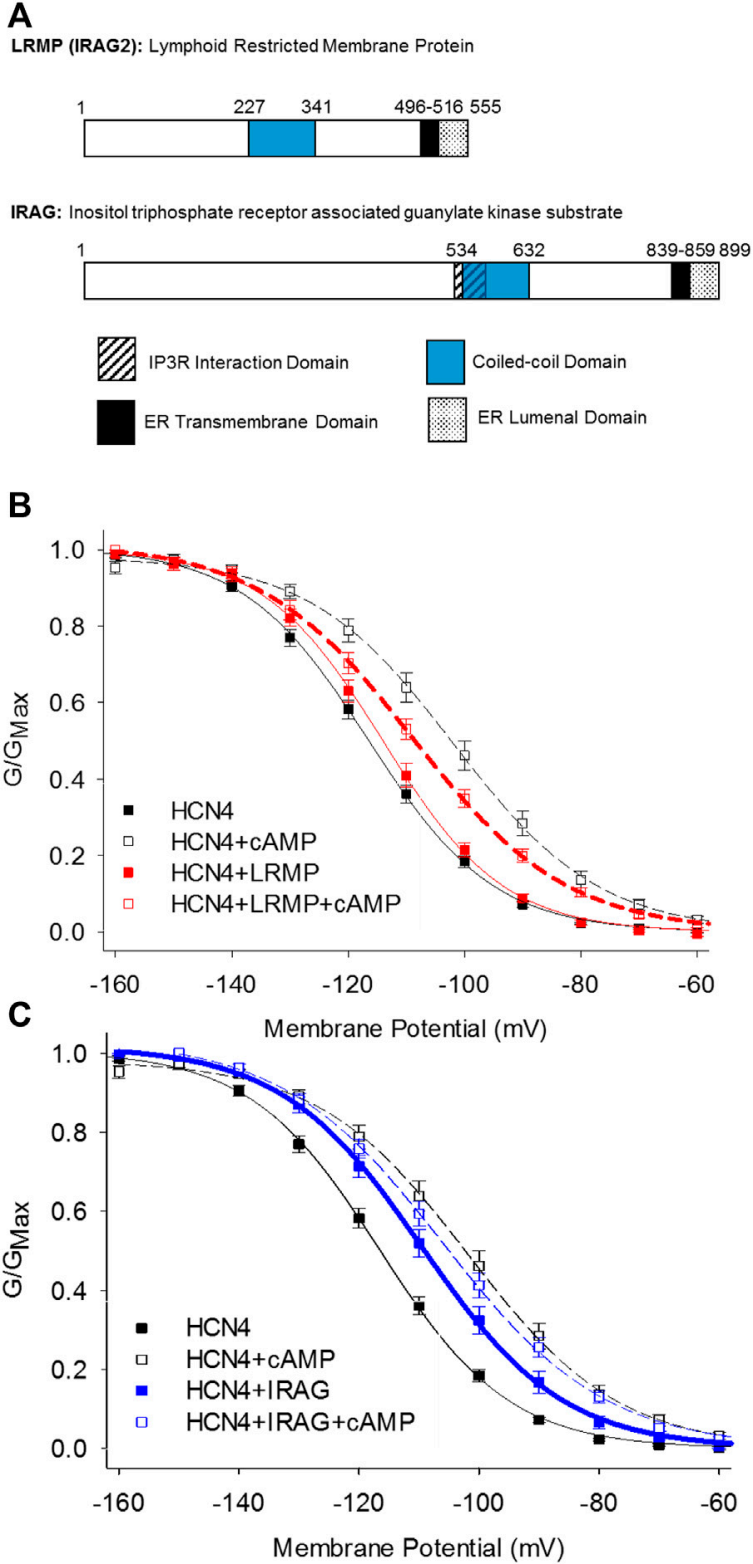
LRMP and IRAG are isoform-specific regulators of HCN4. **(A)** Domain structure of LRMP and IRAG. **(B)** LRMP inhibits cAMP-dependent regulation of HCN4. **(C)** IRAG acts a cAMP mimetic that potentiates HCN4 in the absence of cAMP. From Peters et al., 2020a.

The inositol trisphosphate receptor-associated guanylate kinase substrate proteins, IRAG (IRAG1/Mrvi1) and lymphoid-restricted membrane protein (LRMP/IRAG2), were recently discovered as the first isoform-specific regulators of HCN4 channels (Peters et al., 2020a). These homologous ER-transmembrane proteins possess small ER luminal domains and large cytoplasmic domains containing a coiled-coil motif (Behrens et al., 1994, 1996; Shaughnessy et al., 1999; Figure 3A). Both proteins interact with IP_3_ receptors and IRAG is thought to regulate Ca^2+^ release from the endoplasmic reticulum in response to the NO/cGMP/cGMP Kinase I signaling pathway (Schlossmann et al., 2000; Geiselhöringer et al., 2004; Shindo et al., 2010; Prüschenk et al., 2021).

LRMP and IRAG were identified following an initial observation that the voltage-dependent activation of HCN4 is shifted to more depolarized potentials in a cAMP-independent manner and is not shifted further by exogenous cAMP when the channels are expressed in Chinese hamster ovary (CHO) cells (Liao et al., 2012). This effect is specific to the HCN4 isoform and the CHO cell background; HCN4 responds to cAMP with the canonical shift in voltage-dependence when expressed in HEK293 cells and HCN2 responds to cAMP in both CHO and HEK cells. Although the rabbit isoform of HCN4 may transiently respond to cAMP in CHO cells, the steady-state effects of cAMP on rabbit HCN4 in CHO cells have not been determined (Barbuti et al., 2012). Importantly, this “CHO effect” does not preclude cAMP binding to the CNBD and appears to be mediated by a channel-associated factor because it persists in excised inside-out membrane patches (Liao et al., 2012). Indeed, subsequent co-immunoprecipitation and mass-spectroscopy identified LRMP as an endogenous HCN4 interacting protein in CHO cells and IRAG as an LRMP homologue that also associates with HCN4 (Peters et al., 2020a). When the endogenous LRMP in CHO cells is knocked down using CRISPR, the shift in HCN4 activation is removed and the channels respond normally to cAMP, indicating that LRMP is necessary for the CHO effect (Peters et al., 2020a).

Despite their homology, LRMP and IRAG have opposing effects when co-expressed with HCN4 in HEK293 cells: LRMP reduces the cAMP-dependent shift in the voltage-dependence of HCN4 while IRAG acts as a partial cAMP mimetic, shifting the voltage-dependence to more depolarized potentials in the absence of cAMP (Peters et al., 2020a; Figures 3B,C). Although LRMP and IRAG share an overall domain architecture (Figure 3A), the N-terminus of IRAG is more than 300 residues longer than that of LRMP and the sequences in this region are not conserved. Therefore, the N-terminus is a candidate region that may account for the different effects of LRMP and IRAG on HCN4. The role of the ER transmembrane domains in LRMP and IRAG are not yet clear, but along with the interaction with HCN4 channels on the plasma membrane they tantalizingly hint at the formation of ER-plasma membrane junctions and possibly a coordinated regulation between ER Ca^2+^ release and HCN4 channel activity.

The mechanisms of action of LRMP and IRAG have yet to be determined, but initial observations indicate that they differ substantively from those of TRIP8b. First, unlike TRIP8b which equally impacts all HCN channel isoforms, LRMP and IRAG specifically regulate only HCN4, with no effect on HCN1 or HCN2 (Peters et al., 2020a). Also in contrast to TRIP8b, LRMP and IRAG do not appear to modulate channel expression, as assayed by current density (Peters et al., 2020a). Finally, unlike TRIP8b, which competes with cAMP for binding to the CNBD (Zolles et al., 2009; Santoro et al., 2011; Bankston et al., 2012, 2017; DeBerg et al., 2015), LRMP and IRAG allow cAMP to bind, as evidenced by cAMP-dependent slowing of HCN4 deactivation in the presence of LRMP or IRAG (Liao et al., 2012; Peters et al., 2020a). This observation suggests that LRMP and IRAG may act downstream of cAMP binding, possibly by altering the coupling between the CNBD and the voltage-sensing domain.

The physiological functions of LRMP and IRAG as HCN4-specific modulators suggest a possible role in heart rate regulation. HCN4 is the predominant HCN channel isoform in the sinoatrial node of the heart of all mammals, where it is critical for cardiac pacemaking (Moosmang et al., 1999; DiFrancesco, 2010). Transcripts for both LRMP and IRAG are expressed in the sinoatrial node and IRAG protein is expressed in all regions of the mouse heart (Peters et al., 2020a). Indirect support for contributions of LRMP and IRAG in control of heart rate are suggested by a genome-wide association study linking LRMP to resting heart rate (Eijgelsheim et al., 2010) and a study showing that knockout of IRAG reduces resting heart rate in mice (Geiselhöringer et al., 2004). Interestingly, I_f_ in mouse and rabbit sinoatrial node myocytes is less sensitive to cAMP and opens at more depolarized potentials than heterologously expressed HCN4 channels (Altomare et al., 2003; Larson et al., 2013; Sharpe et al., 2017), consistent with an effect of IRAG expression. However, much additional work will be required to determine whether and how LRMP and/or IRAG impact cardiac pacemaking.

## Tyrosine Kinases

Many types of kinases regulate HCN channels in different systems, including PKA and cGMP-dependent protein kinase II, as well as tyrosine kinases (Wu and Cohen, 1997; Wu et al., 2000; Liao et al., 2010; Hammelmann et al., 2011). While many kinases physically associate with their substrates, such a complex with HCN channels has so far only been demonstrated for the Src tyrosine kinase, which can be co-immunoprecipitated with both HCN2 and HCN4 in ventricular myocytes, mouse brain, and HEK cells, suggesting that it forms a stable complex with HCN channels. However, it is not yet known whether Src kinase interacts directly or indirectly with the channels (Zong et al., 2005; Arinsburg et al., 2006).

Functional regulation of HCN channels by tyrosine kinases has direct physiological consequences in the heart and nervous system. In rabbit sinoatrial node myocytes and dorsal root ganglion neurons, tyrosine kinase activity increases the rate of activation of I_f_ and I_h_, respectively (Wu and Cohen, 1997; Wu et al., 2000; Zong et al., 2005). And Src tyrosine kinase inhibition slows heart rate in Langendorff-perfused mouse hearts (Li et al., 2008). The effects on I_f_ in pacemaker cells are likely due to activation of the HCN4 isoform, however, tyrosine kinase activity also modulates HCN2 (Yu et al., 2004). Indeed, in heterologous expression systems, tyrosine kinase phosphorylation speeds activation of both HCN2 and HCN4 (Zong et al., 2005) *via* phosphorylation of Tyr^476^ in HCN2 and the corresponding Tyr^554^ as well as Tyr^531^ in HCN4 (Zong et al., 2005; Li et al., 2008). In the case of HCN4, tyrosine kinase phosphorylation can restore normal cell surface expression in trafficking-deficient HCN4 D553N mutant channels (Lin et al., 2009).

## KCNE2 (MiRP1)

Among all the HCN interacting partners, perhaps the most ambiguous data concerns the role of the KCNE/MinK-related peptides (MiRPs), which are encoded by the *KCNE* genes. The KCNE family of potassium channel accessory subunits has 5 members, KCNE1 (MinK) and KCNE2-5 (MiRP1-4). The KCNE proteins are composed of a single transmembrane alpha-helix and they can promiscuously assemble with and regulate many different potassium channels in tissues throughout the body. Only KCNE2 has been shown to regulate HCN channels (Yu et al., 2001; Decher et al., 2003), however, the existing information shows a wide range of effects of KCNE2 on HCN channels (Table 1).

**TABLE 1:** Effects of KCNE2 on HCN channel function.

HCN1	Model system	Effects on HCN current	Effects on HCN gating
Yu et al. (2001)	*Xenopus* oocytes	↑ Current	N.S. activation voltage
			↑ activation rate
			N.S. deactivation rate
Lussier et al. (2019)	CHO	↓ Current	N.S. activation voltage
	N.S. activation rate	
	N.S. deactivation rate	
HCN2			
Yu et al. (2001)	*Xenopus* oocytes	↑ Current	N.S. activation voltage
	↑ activation rate	
	N.S. deactivation rate	
Qu et al. (2004)	NRVM	↑ Current	N.S. activation voltage
	↑ activation rate	
	↑ deactivation rate	
Proenza et al. (2002)	CHO	↓ time-dependent current	
	↑ instantaneous current		
Nawathe et al. (2013)	NRVM	N.S.	N.S. activation voltage
	N.S. activation rate	
Lussier et al. (2019)	CHO	N.S.	→ activation voltage
	↑ activation rate	
	N.S. deactivation rate	
HCN4			
Decher et al. (2003)	*Xenopus* oocytes and CHO	↑ Current	← activation voltage
	↓ activation rate	
Altomare et al. (2003)	HEK 293	N.S.	N.S. activation voltage
	N.S. activation rate	
Nawathe et al. (2013)	NRVM	N.S.	N.S. activation voltage
	N.S. activation rate	
	N.S. deactivation rate	
Lussier et al. (2019)	CHO	N.S.	N.S. activation voltage
	N.S. activation rate	
	↑ deactivation rate	

CHO, Chinese hamster ovary cells; NRVM, Neonatal rat ventricular myocytes; N.S, No significant effect; →, Depolarizing shift in voltage-dependence of activation; ←, Hyperpolarizing shift in voltage-dependence.

KCNE2 immunoprecipitates with both HCN1 and HCN2 (Yu et al., 2001; Qu et al., 2004) and increases HCN1, HCN2, and HCN4 current density in *Xenopus laevis* oocytes (Yu et al., 2001; Decher et al., 2003). This interaction involves interactions between the C-terminal regions of each KCNE2 and HCN channel (Decher et al., 2003). In mammalian cells, KCNE2 has been reported to decrease the current density of HCN1 (Lussier et al., 2019), however, it has no impact on HCN2 currents (Lussier et al., 2019), and has been described to either decrease or not alter HCN4 current density in different studies (Altomare et al., 2003; Decher et al., 2003; Lussier et al., 2019). KCNE2 also increased HCN2 currents in transfected neonatal rat ventricular myocytes in one study (Qu et al., 2004) and had no effect on HCN2 or HCN4 currents in another study (Nawathe et al., 2013).

The effects of KCNE2 on HCN channel gating are similarly confusing. Studies have often, but not universally, shown that KCNE2 speeds the activation rates of HCN1 and HCN2 (Yu et al., 2001; Qu et al., 2004; Nawathe et al., 2013; Lussier et al., 2019). However, in the case of HCN4, KCNE2 has been found to decrease (Decher et al., 2003) or not change the rate of activation (Altomare et al., 2003; Nawathe et al., 2013; Lussier et al., 2019). Similarly, KCNE2 has been shown to hyperpolarize the voltage-dependence of HCN4 activation in one study (Decher et al., 2003) and to have no effect in three others (Altomare et al., 2003; Nawathe et al., 2013; Lussier et al., 2019). Importantly, disease-linked KCNE2 variants may alter the effects on HCN channel gating (Lussier et al., 2019). Finally, KCNE2 increases the voltage-independent instantaneous current through HCN2 channels, but a concomitant decrease in the time-dependent current was also noted (Proenza et al., 2002). While the precedents from the IRAGs and TRIP8b raise the possibility of isoform-specific mechanisms of action or different in splice variants of KCNE2, neither of these possibilities has yet been investigated. Ultimately, the ambiguity of these varied studies showcases the need for further studies on HCN channels and KCNE2, with a particular emphasis on mechanisms of action and the effects of KCNE2 expression *in vivo*.

## Other Protein Interacting Partners

While other protein interaction partners have been identified for HCN channels and suggested to play a role in HCN channel function and (patho)physiology (DiFrancesco et al., 2019), many have been the subject of only a few studies. For example, SAP97 colocalizes with HCN4 in sinoatrial node myocytes through a C-terminal PDZ-binding-motif and co-expression decreases HCN4 current density in heterologous expression systems (Peters et al., 2009). However, the significance of this finding has yet to be explored by other researchers.

Caveolin proteins interact with HCN4 through a conserved binding domain in the channel N-terminus (Barbuti et al., 2012) and HCN4 co-localizes with caveolins in heterologous expression systems and isolated sinoatrial myocytes (Barbuti et al., 2007, 2012; Ye et al., 2008). This interaction may be important in localizing HCN4 in the vicinity of β-adrenergic receptors to allow for the sympathetic nervous system to increase sinoatrial myocyte AP firing rate by increasing cAMP (Barbuti et al., 2007). Mutants that disrupt these interactions can decrease HCN4 trafficking and currents (Ye et al., 2008; Barbuti et al., 2012) and alter channel gating (Ye et al., 2008; Barbuti et al., 2012; Campostrini et al., 2017), which may contribute to supraventricular arrhythmias (Campostrini et al., 2017).

The K^+^ channel regulator 1 (KCR1), which regulates ERG and EAG channels (Hoshi et al., 1998; Kupershmidt et al., 2003), has also been shown to interact with HCN2, reducing the current amplitude, and shifting the voltage-dependence of activation to more hyperpolarized potentials in transfected cells (Michels et al., 2008). KCR1 similarly reduces I_f_ in neonatal ventricular myocytes and siRNA knockdown increases the spontaneous beating rate, however this may also be due to its regulation of other channels. KCR1 is expressed at lower levels in adult sinoatrial node tissue, so the physiological implications of this regulation are unclear as of yet (Michels et al., 2008).

HCN channels are also implicated in neurofibromatosis type 1 (NF1) through interactions with the NF1 protein (Omrani et al., 2015). NF1 and HCN1 co-immunoprecipitate from hippocampal lysates and mice lacking the neuron specific NF1 exon 9a exhibit reduced I_h_ in hippocampal parvalbumin neurons and layer 1 interneurons from the visual cortex. This decrease is due at least in part to a hyperpolarized activation of I_h_. Similar results were obtained in a heterozygous NF1 knockout mouse. The decrease in I_h_ is associated with more rapid firing of GABAergic neurons in these mice. Lamotrigine, a Na^+^ channel inhibitor that shifts HCN activation to more depolarized potentials, rescues the visual spatial learning deficits in *Nf1* ^*9a-/9a-*^ mice and motor coordination deficits in *Nf1* ^*+/−*^ mice. The functional effects of NF1 on I_h_ may be directly related to NF1’s role in cAMP production (Tong et al., 2002; Brown et al., 2010), but this has not been confirmed.

Filamin A is an actin cross linking protein that interacts with a non-conserved motif in the HCN1 C-terminus, but does not interact with HCN2 or HCN4 (Gravante et al., 2004; Ramakrishnan et al., 2012). This interaction promotes HCN1 internalization leading to smaller I_h_ currents in neurons (Noam et al., 2014). Unfortunately, the physiological role for this interaction remains unresolved. Similarly, the scaffolding proteins Tamalin, S-SCAM, and Mint2 have all been shown to interact with HCN2 (Kimura et al., 2004), but the consequences of this interaction remain to be studied. Ultimately, open questions remain about the role of many HCN interacting partners.

## Conclusion

HCN channels are crucial regulators of excitability in cells throughout the body. It follows that interacting proteins that modify HCN channel expression, trafficking, and function are also critical for understanding how HCN channels impact excitability. Moreover, the elucidation of mechanisms for HCN channel regulation by these interactors holds potential for the development of new drugs to treat cardiac and neural disease. Ongoing research defining the mechanisms of interaction and how given interacting partners contribute to tissue level excitability remains an exciting avenue of study as further knowledge of HCN regulation also refines our ability to target HCN channels in disease.
